# Prevalence, and health- and sociodemographic associations for visits to traditional and complementary medical providers in the seventh survey of the Tromsø study

**DOI:** 10.1186/s12906-019-2707-1

**Published:** 2019-11-11

**Authors:** Agnete E. Kristoffersen, Ann Ragnhild Broderstad, Frauke Musial, Trine Stub

**Affiliations:** 1 National Research Center in Complementary and Alternative Medicine (NAFKAM), Faculty of Medicine, Department of Community Medicine, UiT The Arctic University of Norway, N-9037 Tromsø, Norway; 2 Centre for Sami Health Research, Department of Community Medicine, UiT The Arctic University of Norway, Tromsø, Norway

## Abstract

**Background:**

Patient-centered culturally sensitive health care (PC-CSHC) has emerged as a primary approach to health care. This care focuses on the cultural diversity of the patients rather than the views of the health care professionals. PC-CSHC enables the patient to feel comfortable, respected, and trusted in the health care delivery process. As users of traditional and complementary medicine (T&CM) rarely inform their conventional health care providers of such use, the providers need to identify the users of T&CM themselves to avoid negative interaction with conventional medicine and to be able to provide them with PC-CSHC. Since the patterns of traditional medicine (TM) use are different to those of complementary medicine (CM), the aim of this study was to investigate the prevalence, and the health- and sociodemographic associations for visits to TM- and CM providers in an urban population.

**Method:**

The data were collected through two self-administrated questionnaires from the seventh survey of the Tromsø Study, a population-based cohort study conducted in 2015–2016. All inhabitants of Tromsø aged 40 or above were invited (*n* = 32,591) and *n* = 21,083 accepted the invitation (response rate 65%). Pearson chi-square tests and one-way ANOVA tests were used to describe differences between the groups whereas binary logistic regressions were used for adjusted values.

**Results:**

The results revealed that 2.5% of the participants had seen a TM provider, 8.5% had seen a CM provider whereas 1% had visited both a TM and a CM provider during a 12-month period. TM users tended to be older, claim that religion was more important to them, have poorer economy and health, and have lower education compared to CM users. We found that more than 90% of the participants visiting T&CM providers also used conventional medicine.

**Conclusion:**

A considerable number of the participants in this study employed parallel health care modalities including visits to conventional, traditional, and complementary medicine providers. To offer patient-centered culturally sensitive health care that is tailored to the patients’ treatment philosophy and spiritual needs, conventional health care providers need knowledge about, and respect for their patients’ use of parallel health care systems.

## Background

Patient-centered health care has emerged as a primary approach to health care. This approach emphasises a partnership between patients and healthcare providers, acknowledges the patients’ preferences and values, and promotes flexibility regarding well-being in the provision of health care [1]. To provide patient-centered health care, health care providers need knowledge about the patients’ health preferences, beliefs, and values. Health preferences and values might vary considerably in populations with mixed culture and ethnicity [2]. Culturally sensitive health care has been described as “health care that effectively responds to the attitudes, feelings, and circumstances of people that share common identifying characteristics (eg. race, religion, language, and socioeconomic status), and health care that patients perceive as being concordant with their cultural values and beliefs” [3, 4]. One important part of patient-centered culturally sensitive health care (PC-CSHC) is to empower the patient [5]. This care focuses on the cultural diversity of the patients rather than the views of the health care professionals [6]. PC-CSHC enables the patients to feel comfortable, respected, and trusted in the health care delivery process [4].

In Norway, PC-CSHC has been emphasised particularly in the Sami population, the indigenous population of Northern Norway, and in immigrants from non-Western countries [7]. The rights of the Sami people in interactions with healthcare are based on national legislation as well as international conventions [8]. In Sami health care, traditional medicine (TM) plays an important role [9, 10]. In addition to the Sami population, Northern Norway is also home to the Kvens, who are the descendants of Finnish-speaking settlers who immigrated from Sweden and Finland to Northern Norway in the 1700 and 1800s [11]. They speak different languages and belong to different cultures [12]. The Sami are indigenous people who traditionally lived as farmers and fishermen or with a semi-nomadic life as reindeer herders [13, 14]. The Kven population, on the other hand, is Finnish immigrants who came to Norway mainly in the 16^th^ and 17^th^ century. They were often farmers settling in areas suitable for farming and forestry [15]. Tromsø is the largest town in Northern Norway as well as a municipality. The population is increasing, partly due to a growing number of people moving from rural areas into the town [16]. The citizens are multi-ethnic. Most of them are Norwegians, but Tromsø also has traditional Sami settlements and a Sami and Kven population who migrated from other areas. Other ethnic groups have also settled in Tromsø mainly due to education and employment at the University, or the University Hospital of Northern Norway [17–20]. Many of these people come from cultures with a strong tradition of using TM.

Even though there are differences in how TM is practiced across cultures, there are also many similarities. TM practices in Northern Norway are influenced by Sami as well as Kven and Norwegian traditions [15, 21]. Due to high costs of conventional medical treatment [15] and lack of medical doctors until recent times [22], the TM systems were well kept in Northern Norway. The Sami and Kven were often members of the Laestadian movement, a conservative Lutheran revival movement that was started in Lapland in the middle of the 19^th^ century, where Sami and Kven preachers traveled around giving sermons in Sami and Finnish [15, 23]. In the Laestadian movement, the Sami and Finnish cultures were valued, making a safe space to continue their TM practice in times when the assimilation process was the official minority policy in Norway [24]. The TM practice today is therefore influenced by Christianity as well as pre Christian nature worship [25–27]. The most commonly used TM modality is healing by prayer (called *reading* as biblical phrases are read over the illness), which is used separately or in combination with tools such as water, herbs, rocks, wool, soil, and steel [15, 27]. One of the specialties of the TM providers in Northern Norway is to stop bleedings. This is used when people are injured and when patients in hospitals suffer from bleedings after childbirth or surgery [15, 27]. Unlike complementary medicine (CM) providers, TM providers are mostly non-professional and non-commercial providers offering their services free of charge or in exchange for small gifts [15, 28, 29]. In this context, CM modalities refer to a broad set of health care practices that are not part of the country’s own tradition nor conventional medicine [30]. Worldwide, CM modalities are used alongside conventional health care [31–33]. In Norway, 36% of the population reported to have used complementary medicine (CM) during the last 12 months; 22% had visited a CM provider, 17% had practiced CM techniques like yoga and meditation, and 10% had used herbal medicine. The most commonly used modalities outside conventional health care were massage therapy (11%), acupuncture (3%), naprapathy (musculoskeletal modality) (3%), and healing (2%) [34]. The practice of TM and CM modalities are equally regulated through Act No. 64 of 27 June 2003 relating to alternative treatment of disease, illness, etc. [35]. The regulation recognizes that T&CM can be provided by both medical and non-medical professionals and within or outside of public health services [36].

An important function of TM providers is to provide support to patients and their families when someone is ill [37]. Both the network around the patients and the patients themselves emphasize the need for health care providers to acknowledge their use of TM, and to facilitate this use for patients who are hospitalized or in nursing homes [37, 38]. Health care personnel report that they facilitate patients who want contact with TM providers and show respect to Christian patients by watching their language. Some even learned the Sami language to better understand their patients and their needs [38]. Previous research regarding TM in Norway has mainly been conducted in rural areas and has demonstrated that 14–50% of the populations studied had used TM [9, 39, 40]. The typical user has low income, Sami affiliation, and physical and mental health challenges [9, 40] compared to non-users of TM. Whereas the users of TM have lower socioeconomic status than the non-users [9], the users of CM have higher education and income compared to the non-users [41, 42]. To offer PC-CSHC, conventional health care providers therefore need to identify the users of TM and CM separately [9]. The use of T&CM is rarely shared with conventional health care providers unless they ask specifically [43, 44] about such use. This non-disclosure increases the risk of interaction between T&CM modalities and conventional treatment [45]. Therefore, the conventional health care providers need information about these users to identify them. The aim of this study was to investigate the prevalence, and the health- and sociodemographic associations for visits to TM and CM providers in an urban population.

## Method

**Fig. 1:**
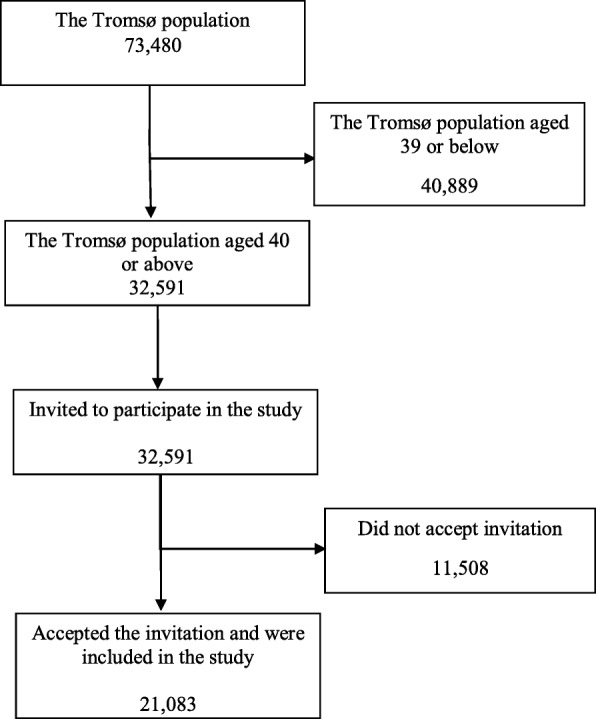
Flow chart of the included participants

The data used in this study is drawn from the 7^th^ survey of the Tromsø Study conducted in 2015–2016 where all inhabitants of the municipality of Tromsø aged 40 or above were invited to participate (*n* = 32,591). *N* = 21,083 accepted the invitation giving a response rate of 65%, Fig. 1. The Tromsø Study is an ongoing longitudinal population-based cohort study among adult inhabitants in the municipality of Tromsø in Northern Norway. The Tromsø study is a collaborative study in the interface between epidemiology and clinical medicine, including a main study that comprises a screening visit, three questionnaires, and several follow-up studies [46]. The first Tromsø study was conducted in 1974.

Tromsø is both a municipality and the largest town in Northern Norway, located 575 km north of the Arctic Circle. Tromsø had 73,480 inhabitants at the time of the study [18], of which approximately 64,500 lived in the town centre. The Tromsø population is increasing, partly due to a growing trend of rural to urban migration in Northern Norway [16]. The Tromsø population is somewhat younger and has higher education compared to the Norwegian average, but is similar in regards to employment rates and income [47].

A postal information letter, followed by an information brochure, and a four-page paper questionnaire (Q1) were sent to all inhabitants of the municipality of Tromsø, aged 40 or above. The postal questionnaire could be returned by mail or the participants could log in with a given user name and password to answer the questionnaire online. Upon login, a questionnaire catalogue was entered with two additional digital questionnaires; a second, more comprehensive questionnaire (Q2), and a body chart with questions about pain, tiredness, and exhaustion. They were also invited to a clinical examination at a given date. When attending the clinical examination, they received a third digital questionnaire with questions about their diet (Q3). Most of the participants completed the survey on site of the clinical examination. Assistance to complete the digital questionnaires was available upon request. A comprehensive clinical examination was then performed and samples of biomarkers such as blood, salvia, and nose and throat samples were collected. The results of these findings will be presented elsewhere. The questionnaires used were not validated as a whole, but consisted of validated parts.

### Measurement used in this study

The data used in this study are based on questionnaire data collected through Q1 and Q2.

### Use of health services (Q1)

The use of *conventional medicine* was based on a *yes* response to either *Have you during the past year visited a general practitioner (GP)?, Have you during the past year visited a psychologist or psychiatrist?, Have you during the past year visited a physiotherapist?* or *Have you during the past year been admitted to a hospital?*

The use of TM was based on a *yes* response to: *Have you during the past year visited a traditional healer (helper, “reader”,* etc.*?).* The use of CM was based on a *yes* response to either of the two questions: *Have you during the past year visited an acupuncturist?* or *Have you during the past year visited a CM provider (homeopath, reflexologist, spiritual healer,* etc.*?).* The participants classified as users of both TM and CM providers were participants answering *yes* to both *Have you during the past year visited a traditional healer (helper, “reader”,* etc.*?),* and *Have you during the past year visited an acupuncturist?* or *Have you during the past year visited a CM provider (homeopath, reflexologist, spiritual healer,* etc.*?).* In the analyses, the categories *Visits to TM providers, visits to CM providers,* and *visits to TM as well as CM providers* were mutually exclusive.

The respondents answering yes to either of these questions were in addition asked to report the number of times they had seen the therapists during the last year.

### Self-reported health

Self-reported health was measured through two variables. The first variable was categorical and collected in Q1: *How do you in general consider your own health?* with the five response categories *very bad, bad, neither good nor bad, good*, and *excellent*. These response options were re-organized into three categories: *Bad* (very bad, and bad), *Neither good nor bad,* and *Good* (good, and excellent). The second variable was continuous and from Q2: *We would like to know how good or bad your health is today. This scale is numbered from 0 to 100. One hundred means the best health you can imagine. Zero means the worst health you can imagine. Please insert a number between 0 and 100.*

### Age, income, ethnicity

The measure of age was a continuous variable measuring the participant’s age per 31.12.2015.

Income was measured by 7 response categories in Q1 (Less than NOK 150′/EUR 15′, NOK 150′-250′/EUR 15′-25′, NOK 251–350′/EUR 25.1′-35′, NOK 351′-450′/EUR 35.1′-45′, NOK 451′-550′/EUR 45.1′-55′, NOK 551′-750′/EUR 55.1′-75′, NOK 751′-1000′/EUR 75.1′-100′ and more than NOK 1000′/EUR 100′) which was re-categorized into: *Less than NOK 450′/EUR 45′*, NOK *450′-750′/EUR 45′-75′* and *more than NOK 750′/EUR 75′*. The question *How would you evaluate your finances?* was measured through 5 response categories (*Very good*, *good*, *average*, *difficult,* and *very difficult*) and merged into the following three categories: *Good* (very good, and good), *average,* and *difficult* (difficult, and very difficult).

Ethnicity was measured by the Q2 question: *What do you consider yourself as (check all that apply)* with the 4 response categories*: Norwegian, Sami, Finnish/Kven,* and *Other*. Ethnicity can be defined in different ways, depending on the criteria. In this study, *Norwegian* included participants identifying themselves solely as Norwegians. *Sami/Kven affiliation* included all who defined themselves as Sami and/or Kven regardless of other ethnic affiliations. “*Other”* consisted of participants checking only for “*Other”*.

All response categories of the Q2 question *What is the importance of religion in your life?,* and the Q1 questions *Do you live with a spouse/partner?,* and *What is the highest level of education you have completed?* are presented in Table 1.

**Table 1:** Basic characteristics of the total sample and of the participants visiting TM providers, CM providers, and TM as well as CM providers

	The total population		No visits to T&CM providers		Visits to TM providers		Visits to CM providers		Visits to both TM and CM providers		*p*-value*
	% (*n* = 21,083)		% (*n* = 18,977)		% (*n* = 324)		% (*n* = 1580)		% (n = 202)		
**1. Self-reported health (scale 0–100)**											
Mean (SD)	76.1 (16.32)		76.5 (16.10)		68.6 (17.73)		73.1 (17.31)		66.9 (18.57)		< 0.001^b^
Missing	416		377		10		21		8		
**2. Self-reported health**^**1**^											
Good	68.4	14,290	69.6	13,094	45.0	144	61.2	957	47.7	95	<0.001^a^
Neither	26.1	5450	25.3	4762	42.2	135	30.5	478	37.7	75	
Bad	5.6	1163	5.1	963	12.8	41	8.3	130	14.6	29	
Missing		180		158		4		15		3	
**3. Use of other health care last year**											
Seen a GP	80.5	16,852	79.3	14,935	92.9	301	91.2	1432	91.1	184	0.583^a^
Missing		153		144		0		9		0	
Number of visits to GP Mean (SD)	3.46 (3.61)		3.32 (3.49)		5.74 (5.84)		4.24 (3.82)		5.01 (4.75)		< 0.001^a^
Missing		1619		1457		37		107		18	
Hospitalized	11.0	2297	10.6	1989	24.1	77	12.7	199	15.9	32	< 0.001^a^
Missing		209		190		5		12		1	
**4. Age**											
Mean (SD)	57.3 (11.42)		57.46 (11.44)		59.3 (12.94)		55.4 (10.75)		55.8 (10.67)		< 0.001^b^
40–59 years	59.1	12,467	60.1	11,094	53.9	172	69.3	1069	67.3	132	< 0.001^a^
60 years and above	38.2	8052	39.9	7369	46.1	147	30.7	473	32.7	64	
**5. Gender**											
Men	47.5	10,009	49.0	9294	38.6	125	32.7	516	36.6	74	0.085^a^
Women	52.5	11,074	51.0	6983	61.4	199	67.3	1064	63.4	128	
**6. Living with a spouse/partner**											
Yes	76.8	15,283	77.1	13,814	72.4	213	76.3	1137	64.3	119	0.001^a^
No	23.2	4609	22.9	4108	27.6	81	23.7	354	35.7	66	
Missing		1191		1055		30		89		17	
**7. Household income**^**2**^											
< NOK 451′/ € 45’	22.5	4545	22.1	4019	37.9	114	22.0	336	39.8	76	< 0.001^a^
NOK 451′-750′/€ 45′-75’	29.2	5884	29.1	5269	33.9	102	29.6	452	31.9	61	
> NOK 750′/€ 75’	48.3	9756	48.9	8880	28.2	85	48.3	737	28.3	54	
Missing		898		809		23		55		11	
**8. How will you evaluate your finaces**^**3**^											
Good	70.3	14,554	71.0	13,246	54.4	170	66.4	1035	53.1	103	<0.001^a^
Average	26.2	5426	25.7	4792	36.2	113	28.9	451	36.1	70	
Difficult	3.6	737	3.3	614	9.3	29	4.7	73	10.8	21	
Missing		366		325		12		21		8	
**9. Years of education**											
Primary school	23.2	4796	22.9	4263	39.2	123	22.5	350	30.3	60	<0.001^a^
Secondary school	27.8	5756	27.6	5147	26.8	84	30.0	467	29.3	58	
College/university less than 4 years	19.4	4008	19.3	3603	16.2	51	20.5	318	18.2	36	
College/university 4 years or more	29.7	6145	30.2	5625	17.8	56	27.0	420	22.2	44	
Missing		378		339		10		25		4	
**10. Ethnicity**											
Norwegian	92.4	19,040	92.6	17,180	86.8	270	91.9	1424	86.5	166	<0.001^a^
Sami/Kven	4.2	857	3.9	725	12.5	39	4.7	73	10.4	20	
Other	3.4	707	3.5	647	0.6	2	3.4	52	3.1	6	
Missing		479		425		13		31		10	
**11. Importance of religion**											
Very important	8.3	1714	7.6	1414	36.8	113	9.7	150	19.0	37	<0.001^a^
Somewhat important	48.6	10,019	48.4	8969	48.2	148	51.5	797	5.8	105	
Not important	43.0	8862	44.0	8163	15.0	46	38.8	600	27.2	53	
Missing		488		431		17		33		7	

^a^Pearson’s chi-square test; ^b^One-Way ANOVA test; ‘1000; ^1^The differences remained when adjusting for age (*p* < 0.001), education (*p* < 0.001), and income (*p* < 0.001); ^2^The differences remained when adjusting for age (*p* = 0.015), self-reported health (*p* = 0.001), and living with a spouse/partner (*p* = 0.009); ^3^The differences remained when adjusting for age (*p* < 0.001) and self-reported health (*p* < 0.001), but not when adjusted for living with a spouse/partner (*p* = 0.803)

Variable names are presented in bold

*The p-value refers to the difference between the following three groups: Visits to TM providers, Visits to CM providers, and Visits to both TM and CM providers

### Statistics

We used Pearson chi-square tests and one-way ANOVA tests to describe the basic characteristics of the participants and to calculate differences between the users of TM, CM, and the users of both TM and CM (Table 1). For adjusted values (presented in the text only), binary logistic regressions were used. SPSS for Windows (version 24.0, SPSS, Inc., Chicago, IL) was used for all the analyses. The significance level was set at *p* < 0.05.

## Results

### Prevalence of use

**Fig. 2:**
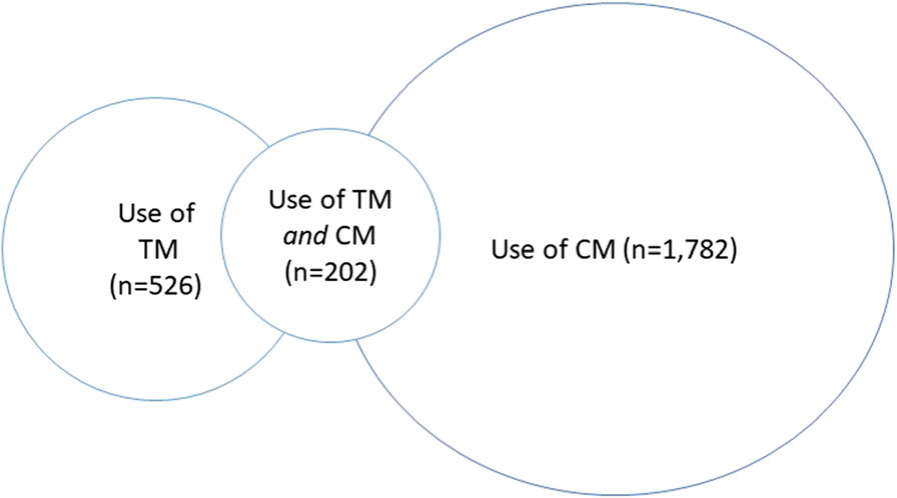
The participants divided in the studied groups

Of the participants, *n* = 17,303 (82.1%) had used conventional medicine, *n* = 16,852 (80.5%) reported to have seen a GP with a mean number of 3.46 visits during the last year (SD 3.61), and *n* = 2297 (11%) had been hospitalized. T&CM providers were visited by 2106 participants (10%); *n* = 526 (2.5%) had visited a TM provider, *n* = 1782 (8.5%) had visited a CM provider whereas *n* = 202 (1%) had visited TM as well as CM providers (Fig. 2). The majority of the participants who had visited T&CM providers had also used conventional health care (94.2%, *n* = 1974), with only small differences between the users of TM and CM (*p* = 0.326).

### Associations for use of T&CM

We found that age, single or living with a partner, household income, finances, educational level, ethnicity, importance of religion, self-reported health, and hospitalization associated differently for participants visiting TM providers compared to participants visiting CM providers. We did not find significant differences regarding gender and whether the participants had consulted a GP. Women were more likely to have seen both TM- and CM providers than men (Table 1, point 5 (1:5).

### Sociodemographic associations

The participants visiting TM providers were on average 3.9 years older than those visiting CM providers, and more likely to be 60 years or older (46.1% vs 30.7% respectively, *p* < 0.001, Table 1:4). They had lower education (primary school only: 39.2% versus 22.5%, *p* < 0.001, Table 1:9) and were less likely to live with a spouse/partner (72.4% versus 76.3%, *p* < 0.001, Table 1:6). The participants visiting TM providers had lower household income (*p* < 0.001, Table 1:7) and evaluated their finances as poorer (*p* < 0.001, Table 1:8) compared to those visiting CM providers. The differences in household income remained when adjusted for age (*p* = 0.015), health (*p* = 0.001) and whether the participants lived with a spouse/partner or not (*p* = 0.009, Table 1:7). This was also the case for the differences regarding the participants’ financial situation, which remained when we adjusted for age (*p* < 0.001) and self-reported health (*p* < 0.001, Table 1:8). When we adjusted for living with a spouse/partner, there were no longer significant differences between those who had visited TM providers and those who had visited CM providers regarding their financial situation (*p* = 0.803, Table 1:8).

### Health related associations

The participants visiting TM providers reported in general poorer health than those visiting CM providers. On a scale from 0 (as bad as it could be) to 100 (as good as it could be) the participants visiting TM providers had a mean score of 68.6 compared to 73.1 among those consulting a CM provider (*p* < 0.001, Table 1:1). Poor health was reported by 12.8% of the participants visiting TM providers compared to 8.3% of those visiting CM providers (*p* < 0.001, Table 1:2). The significant differences between those who had consulted a TM provider and those who had consulted a CM provider remained when adjusted for age (*p* < 0.001, Table 1:2), education (*p* < 0.001, Table 1:2), and income (*p* < 0.001, Table 1:2). The highest number of participants with poor health (14.6%) was identified among the participants who had seen both TM- and CM providers (Table 1:2).

The participants who had visited a TM provider reported more frequent visits to their GP than those who had visited a CM provider (a mean of 5.74 times compared to 4.24, *p* < 0.001, Table 1:3). They were also more likely to have been hospitalized than those who had consulted a CM provider (24.1% versus 12.7%, *p* < 0.001, Table 1:3).

### Importance of religion

Participants visiting T&CM providers reported religion to be a more important part of their lives than those who did not. Most important was religion to those who had visited a TM provider as 36.8% reported religion to be very important in their lives. Only 9.7% of those who had consulted a CM provider reported the same (*p* < 0.001). (Table 1:11).

### The relevance of ethnicity

Most of the participants visiting TM providers (86.8%, *n* = 270), CM providers (91.9%, *n* = 1424), and TM as well as CM providers (86.5%, *n* = 166) defined themselves as Norwegians (Table 1:10). Whereas the Sami/Kven were most likely to have seen TM providers, the Norwegians and the participants of other ethnicities were most likely to have seen CM providers (*p* < 0.001, Table 1:10).

## Discussion

We found that 10% of the participants had visited T&CM providers; 2.5% had visited TM providers and 8.5% had consulted CM providers. One percent had been in contact with both TM and CM providers during a 12-month period. This study demonstrates that more than 90% of the participants who had seen T&CM providers employed parallel health care modalities by adding conventional medicine to their use of T&CM. This is in accordance with previous findings in Northern Norway [9, 33, 48]. This underlines the need for PC-CSHC and for conventional health care personnel to be able to recognize the users of T&CM to provide this. The visitors to TM providers tended to be older, have poorer economy and health, and lower education compared to those who had seen CM providers. They also claimed that religion was a more important part of their lives.

Even though conventional medicine is the officially approved medical system in Norway, many people choose additional modalities to improve their health or as a consolation in a challenging health situation [48–50]. Our study supports earlier findings showing that TM exists along with CM outside the official health care system [9, 51]. Patients seem to be active and tailor their own holistic health care to meet medical, spiritual, as well as cultural needs [52]. Access to T&CM and conventional health care may allow patients to make their own choices with regard to cultural validation, a great sense of control of the disease process, symptomatic cure, a better understanding of its multi-dimensional causation, and the benefit of two or more expert opinions (GP and T&CM providers). This is a form of medical pluralism implying that in any society, patients may resort to different kinds of treatment modalities, even where these have mutually incompatible explanations for the illness [53]. When patients want to see T&CM providers within a hospital or a nursing home setting, they might need other facilities than those provided for conventional health care. To be able to provide patients with PC-CSHC, conventional health care workers need information about the patients’ needs and preferences in this regard. As many patients are afraid to be stigmatized and regarded as more diseased if they openly share their view of their illness and visits to T&CM providers [38, 54, 55], the initiative to discuss this must lie with the conventional health care providers. Most conventional health care providers lack this initiative [44]. One of the most common reasons for this is lack of knowledge about T&CM modalities and the philosophy base of these modalities [44, 45]. To be more willing to discuss this with patients, conventional health care providers need to increase their knowledge about the users of these modalities. By doing so, they can reduce the gap between patient and provider [56], and strengthen patient-centered communication [44].

Our findings suggesting that people consulting TM providers find religion more important, have poorer health, and lower income than those who do not, are in accordance with findings in a study conducted in areas with a mixed Sami and Norwegian population (The SAMINOR 1 Survey) in 2003–2004 [9]. The fact that these findings are based on data collected in two different populations (urban/mainly rural) and with a time difference of 12 years strengthens the validity of those associations. Lower income and education among the users of T&CM were also found in a recent review mapping the T&CM use in Sub-Saharan Africa [57]. Our findings of lower educational level were, however, not in accordance with the findings in the SAMINOR 1 Survey, which may be due to lower educational level in general among their participants (33% vs. 49% with university education). The same survey [9] found that most participants who had seen TM providers identified themselves as Sami. In our study, these participants identified themselves mainly as Norwegians. The main reason for this is probably the much lower proportion of Sami participants in the present study. This underlines, however, that urban Norwegians, Sami, and Kven visit TM providers, though to a less degree than what was found in the SAMINOR 1 Survey.

A higher use of TM was also found in the smaller town Alta in 1975 where 42% of the participants, regardless of ethnicity, had used TM [39], and among Alaska Natives where 46% had used TM [58]. The lower use of TM found in the present study may be due to the fact that the participants were recruited outside a health care setting and therefor consisted of mostly healthy participants. Also the fact that only visits to TM providers were asked for and not over all use of TM might have influenced the lower number of TM users. We also asked for use of TM within a time frame of 12 months while the other studies asked for lifetime use of TM.

The lower number of participants with ethnic minority background might also have influenced the findings, as the use of TM has been associated with indigenous ethnic identity [9, 59]. The higher proportion of participants from rural areas included in some of the other studies might also have played a role. However, the differences are not likely to indicate a decrease over time as similar use of TM was found in the 4^th^ Tromsø study conducted in 1994–1995 [60].

Our findings indicating that the use of TM is associated with older age is in accordance with findings in the US [59] and South Africa [61], but not with the Norwegian SAMINOR 1 Survey. The discrepancy with the SAMINOR survey may be attributed to the more common use of TM among the Sami [9, 39, 40], and the fact that TM is regarded as more mainstream health care in the Sami areas [37, 38]. Therefore, young people might be likely to use TM to a greater extent. Moreover, the lifetime use of TM reported in the SAMINOR 1 Survey may have included users who do not struggle with health complaints at present, but have used TM sometimes in the past, for instance as children.

The lower number of participants who reported to have visited TM providers compared to CM providers, and the higher ages, and poorer health reported by those who had seen TM providers, might be because visits to TM providers are made when illness occurs [10]. People seeing CM providers are also known to do so for disease prevention, well-being, and to improve the immune system as well as treat illness and chronic complaints [62, 63].

The reason why those who had seen TM providers regarded religion to be such an important part of their lives may be because TM rituals often contain prayer and biblical phrases, and that TM is often used in a Christian context such as within the Laestadian movement [38]. Henriksen suggested that the use of TM is an expression of everyday faith, similar to how evening prayer is used when someone is ill [64]. Higher religious awareness was also found among those who had seen CM providers, however to a lower degree than for those who saw TM providers. People visiting T&CM providers seem to have a holistic world-view, including the belief that humans are spiritual beings [65], as found earlier in other countries [57, 59, 66].

A stronger association was found between hospitalization/poor health and visits to TM providers than what was the case for CM providers. Larsen et al. [37, 38] found that when patients were hospitalized, the patients’ network contacted the TM providers and asked them to send healing to their hospitalized relatives and include the medical diagnoses in their rituals. She also found that the TM providers gave the patients prayer cloths (pieces of fabric with printed prayers) to wear when they were seriously ill, and that the patients were worried that these cloths should disappear when their hospital clothes were sent with the laundry. Consequently, the hospital personnel needed to be aware of their patients’ use of TM and that prayer cloths might be part of the treatment [37, 38]. It seems like the tradition of contacting CM providers when hospitalized is not as strong. Even though many hospitals in Norway offer CM modalities [67], this might not be the case in the university hospital in Tromsø. Another reason for this might be that the patients’ network has a tradition for contacting TM providers on behalf of hospitalized relatives rather than CM providers [37].

The fact that TM providers, contrary to CM providers offer their services free of charge or in exchange for small gifts, makes TM a good treatment option for people with limited financial resources. The reason the TM providers do not charge money for their services is because they believe that God, as a gift of grace, gave healing abilities to them: “*Heal the sick, raise the dead, cleanse lepers, cast out demons. You received without paying; give without pay.*” (The Holy Bible, Matthews 10, 7–8). They believe that they lose their ability to heal if they charge money for their services. For the same reason, many TM providers show disrespect for modern healers who charge money for their services [15, 27]. Many TM providers are members of the Laestadian movement and strong believers of this practise deeply rooted in their culture [25, 64, 68].

### Strengths and limitations

The main strength of this study is the large sample size (*n* = 21,083), the rather high response rate (65%), and the unselected sample of the target population where all residents aged 40 or above in the Tromsø municipality were invited. Even though population studies are considered an excellent source for research [46], the results should be interpreted in light of some limitations. One limitation is that the cross sectional design of the study does not provide information on causality to any of the associated factors found [69]. The validity of self-reported data can also be questioned, although the agreement between self-reported data and registered health care utilization is generally high [70], and that sensitive information like visits to T&CM providers might be easier to report in self-administered questionnaires [71]. Missing responses to single questions might also have influenced the overall findings, even though the missing responses were generally low. The fact that all the participants were 40 years or older limits the findings to middle aged and elderly people. Also the “etc.” used in the questions *Have you during the past year visited a traditional healer (helper, “reader”,* etc.*?)* and *Have you during the past year visited a CM provider (homeopath, reflexologist, spiritual healer,* etc.*?)* might be confusing. We think, however, that we have listed enough examples of words for traditional medical providers for the participants to understand the question, and likewise for complementary medical providers. In the Norwegian wording, the difference between the traditional healer (hjelper, “læser” etc) and spiritual healer (healer) should be clear.

### Implementation of the findings

To identify T&CM users and provide them with the best PC-CSHC, it is important for health care personnel to improve their knowledge and understanding of the users of T&CM, a group that expresses additional health care needs compared to the non-users. To facilitate for visits from T&CM providers in hospitals and nursing homes, to open-minded welcome T&CM providers, and discuss T&CM use with patients in a non-judicial way, are ways of providing PC-CSHC for patients who wish to add T&CM to their treatment program. To be able to discuss the patients’ use of T&CM, health care providers might need to increase their knowledge of the most commonly used therapies in their area. In Northern Norway, several health care workers grew up in areas where TM providers were a natural part of their upbringing. They report that they call TM providers on behalf of the patients, and occasionally take part in TM rituals initiated by the patients [38]. TM rituals often combine healing prayers and tools [72]. Steel is a material often used, placed where the patient hurts or to scare demons away [38]. Prayer cloths fastened to hospital shirts with safety pins should not follow the shirts to the laundry [38]. As Sami people more often than other groups add TM to their health care [9], knowledge from this study might be useful for conventional health care providers who wish to provide PC-CSHC in other areas in Norway, Sweden, Finland, and Russia with a Sami population. Knowledge of different health- and sociodemographic associations for visits to TM providers and CM providers might be useful for other researchers in the field researching associations for the use of T&CM.

As use of T&CM can interact with conventional health care, health care providers need to be extra aware of T&CM use in patients receiving treatment that can be effected by such use. Despite the fact that medical personnel have an ethical responsibility to discuss the use of T&CM with their patients [73], few do so on a regular basis [38, 44]. As neither patients nor conventional health care providers seem to take initiative to discuss this topic [45], we urge conventional health care providers to take this initiative and make sure that the patients’ use of T&CM is described in the patients’ medical record. This study revealed that the majority of those who visited T&CM providers also sought help from conventional health care providers. The T&CM providers should, however be aware of the small amount of their patients who do not use conventional health care, and try to identify them to discuss this matter. We also urge the T&CM providers to map use of other T&CM modalities used by their patients to reveal possible negative interactions of the different treatment modalities the patient receive.

The differences between people visiting TM providers and people visiting CM providers found in this study show that combining the associations for TM and CM use could undermine the true associations for TM as well as CM. To be able to offer PC-CSHC, conventional health care providers should ask patients about their use of TM and CM separately. Especially, when consulting older men and women with severe health challenges, who are not considered main users of CM modalities.

Information from the present study may contribute to improving this knowledge and hence the quality of the Norwegian public health service.

### Further research

As the present study only describes visits to TM- and CM providers in a small area among middle aged and elderly people, further research should focus on comparison in areas where TM is more commonly used as well as in younger populations. Measurements of lifetime use of TM and CM might give different associations than the use limited to the last 12 months. As the TM practiced in Northern Norway is influenced by the Sami, the study should be repeated in other countries hosting Sami populations, namely Sweden, Finland, and Russia to see if similar patterns may be found in these countries.

## Conclusion

To meet individual health care needs, the participants in this study employed parallel health care modalities including conventional, traditional, and complementary medicine. To offer PC-CSHC tailored to patients’ treatment philosophy and spiritual needs, it is important that health care personnel have knowledge about their patients’ use of parallel health care system.

## Data Availability

The raw dataset is not available due to Norwegian privacy regulations. Applicants for any data must be prepared to conform to Norwegian privacy regulations.

## Abbreviations

CM: Complementary Medicine
EUR: Euro
NOK: Norwegian Kroner
PC-CSHC: Patient-Centered Culturally Sensitive Health Care
REK: Regional Committee of Medical and Health Research Ethics
SD: Standard Deviation
T&CM: Traditional and Complementary Medicine
TM: Traditional Medicine
UiT: University of Tromsø
